# Septipyridines as conformationally controlled substitutes for inaccessible bis(terpyridine)-derived oligopyridines in two-dimensional self-assembly

**DOI:** 10.3762/bjnano.2.46

**Published:** 2011-07-26

**Authors:** Daniel Caterbow, Daniela Künzel, Michael G Mavros, Axel Groß, Katharina Landfester, Ulrich Ziener

**Affiliations:** 1 Institute of Organic Chemistry III/Macromolecular Chemistry; 2 Institute of Theoretical Chemistry, University of Ulm, Albert-Einstein-Allee 11, D-89081 Ulm, Germany; 3 Max Planck Institute for Polymer Research, Ackermannweg 10, D-55128 Mainz, Germany

**Keywords:** oligopyridines, self-assembled monolayer, STM

## Abstract

The position of the peripheral nitrogen atoms in bis(terpyridine)-derived oligopyridines (BTPs) has a strong impact on their self-assembly behavior at the liquid/HOPG (highly oriented pyrolytic graphite) interface. The intermolecular hydrogen bonding interactions in these peripheral pyridine units show specific 2D structures for each BTP isomer. From nine possible constitutional isomers only four have been described in the literature. The synthesis and self-assembling behavior of an additional isomer is presented here, but the remaining four members of the series are synthetically inaccessible. The self-assembling properties of three of the missing four BTP isomers can be mimicked by making use of the energetically preferred N–C–C–N transoid conformation between 2,2'-bipyridine subunits in a new class of so-called septipyridines. The structures are investigated by scanning tunneling microscopy (STM) and a combination of force-field and first-principles electronic structure calculations.

## Introduction

Two-dimensional molecular self-assembly is a common approach to build up surface-supported nanostructures [1–2]. Appropriately-directed intermolecular interactions are required to guarantee nearly perfect ordering of these monolayers. Hydrogen bonding interactions serve this purpose: They are directed, of intermediate strength, and adjustable [3–4]. The bis(terpyridine)-derived oligopyridines (BTPs), are large polyaromatic molecules with *C*_2_*_v_* symmetry, are well established compounds, and are known to self-assemble [5] at the liquid/HOPG [6–11] and at the gas/solid interface [12–14]. Self-assembly also occurs under electrochemical conditions [15], and the molecules assemble into a broad variety of two-dimensional (2D) structures based on weak intermolecular C–H^…^N hydrogen bonds. Due to the directionality of the hydrogen bonds, the relative orientation of the peripheral pyridine rings has a strong impact on the 2D structure; in contrast, the substrate has a minor influence on the pattern formation [12]. Besides the preset constitution of each BTP molecule, different conformations are possible, which might subsequently lead to different monolayer structures. The influence of the conformation of adlayer molecules on the 2D structures formed is described in several examples in the literature. Most often, flexible substituents on more rigid core units lead to different conformers, which self-assemble in different 2D structures as shown, e.g., for porphyrin [16], phthalocyanine [17], and quinacridone derivatives [18], N,N-diphenyl oxalic amide [19], bithiophene–fluorenone conjugated oligomers [20], a 2,6-di(acetylamino)pyridine substituted conjugated module [21], and a molecular hexapod having a benzene core and six oligo(*p*-phenylene vinylene) legs [22]. The conformers can have an impact on the expression or suppression of chirality of the supramolecular structures [23–25], and sometimes extrinsic factors, such as the presence of guest molecules [26–27], or the pH value [28–29], trigger the formation of certain self-assembled conformers. It is known that for 2,2'-bipyridine, the transoid conformation of the N–C–C–N unit is preferred because of dipole–dipole interactions [30].

**Figure 1:**
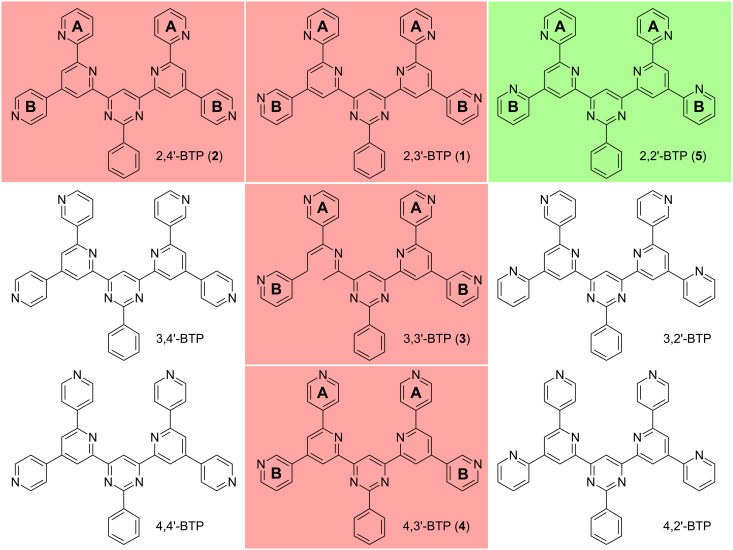
Nine possible constitutional BTP isomers with the four already described in literature (in red) [6–7] and the one newly synthesized as described in this paper (in green).

Due to the *C*_2_*_v_* symmetry of the BTP molecules, nine constitutional isomers are possible by varying the connection between the four peripheral and the two central pyridine moieties (Figure 1). Until now, four compounds of this series have been described in the literature, including details of their 2D self-assembly [6–7]. The various isomers with their different peripheral pyridine ring orientations originate from the orientation of the pyridine rings in the different diazachalcone precursor molecules.

Here we present the synthesis of a fifth BTP isomer, 2,2'-BTP (**5**), and its self-assembly properties at the liquid/HOPG interface. Additionally, a new class of oligopyridines comprising seven pyridine units and one phenyl substituent, the so-called phenylseptipyridines (PhSpPy), is presented. The PhSpPys display the same symmetry, shape, and size as the BTPs and can be regarded as substitutes for the inaccessible BTPs in terms of their 2D self-assembly behavior as shown by STM.

## Results and Discussion

### Synthesis

**Scheme 1:**
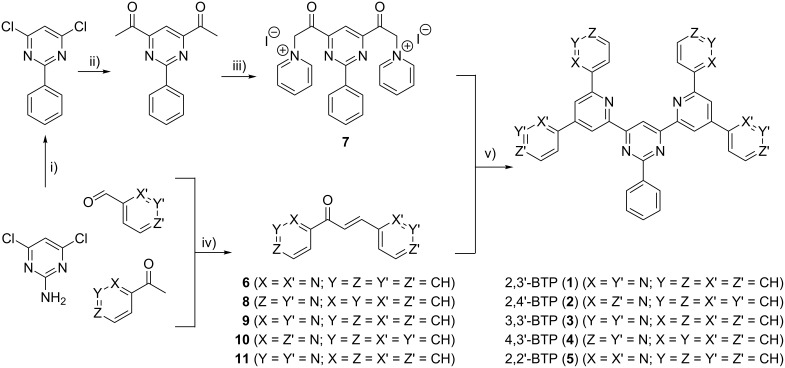
Synthetic pathway to BTPs (**1**–**5**). i) Cu_2_O, isoamylnitrite, benzene, 100 °C, 3 h; ii) (1-ethoxy)-vinyl-tributylstannane, Pd(PPh_3_)_4_, DMF, 20 h reflux, acetone, HCl; iii) iodine, pyridine, 100 °C, 4 h; iv) MeOH, NaOH; v) NH_4_OAc, MeOH reflux.

The synthesis of the BTPs is based on a double ring-closure reaction corresponding to the Kröhnke synthesis (Scheme 1) [31]. One component is the bipyridine-substituted unsaturated ketone (diazachalcone). The binding sites of the pyridine rings determine the orientation of the peripheral nitrogen atoms in the final BTPs. The diazachalcones were obtained from the condensation between the corresponding pyridylaldehyde and the acetylpyridine. Unfortunately, the only diazachalcones accessible are those that are derived from 2-acetylpyridine and/or 3-pyridylcarbaldehyde. We attribute this finding to the electron withdrawing effect of the pyridine rings, which is most pronounced in the *ortho*-position; this effect stabilizes the carbanion of the 2-acetylpyridine more effectively than the carbanions derived from the two other isomers. The unsuccessful trials to obtain the missing diazachalcones delivered mainly the double aldol adducts; the presence of these products suggests high reactivity of the aldehyde, which facilitates the attack of a second acetyl compound instead of elimination of water, a key step in the condensation leading to the formation of the BTP compounds. Thus, only the least reactive aldehydes, namely the *meta*-isomers, led to the desired diazachalcones. Consequently, by the reaction of 2,2'-diazachalcone (**6**) with the bis(pyridinium) salt (**7**), a fifth bis(terpyridine)-derived oligopyridine, 2,2'-BTP (**5**), could be added to the already known four isomers (**1**–**4**).

**Figure 2:**
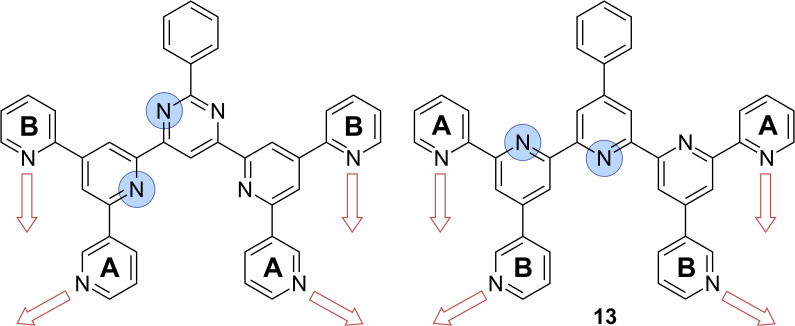
The synthetically unavailable 3,2'-BTP (left), which is representative of the other inaccessible BTPs, is hypothetically derived from the unavailable 3,2'-diazachalcone. The corresponding 2,3'-PhSpPy (**13**) (right), derived from the available 2,3'-diazachalcone, should adopt the transoid N–C–C–N conformation [30] in the coplanar adsorbed state and thus the same orientation of the peripheral pyridine units.

The known BTP isomers show a large variety of self-assembled 2D structures [7]. It is expected that the four missing isomers should also form interesting 2D structures; however, since they are synthetically inaccessible, a new molecular design is required to mimic the structure of the BTPs in order to study the 2D self-assembly properties of the missing isomers. The essential factors, which determine the self-assembled structure of the known BTPs are the intermolecular C–H^…^N hydrogen bonds, which are governed by the relative orientation of the peripheral pyridine rings. Thus, we seek *C*_2_*_v_*-symmetric oligopyridines with 3,4'-, 3,2'-, 4,4'-, and 4,2'-orientation of the nitrogen atoms, with the first number indicating the connection of the A pyridine rings and the second the B pyridine rings (see Figure 1). The corresponding required diazachalcones are not synthetically available, but the 4,3'-, 2,3'-, and 2,4'-diazachalcones (**8**–**10**) are. As the BTPs in the 2D structures are essentially coplanar, they adopt the transoid N–C–C–N conformation between the pyrimidine and the central pyridine units, similarly known for 2,2'-bipyridine [30]. If the core unit of the BTPs is exchanged for a pyridine moiety, the opposite conformation should be preferably formed. Finally, the corresponding phenylseptipyridines prepared from the available 2,4'-, 2,3'-, 2,2'-, 3,3'-, and 4,3'-diazachalcones (**6, 8–11**) should adopt the same conformation as the 4,2'-, 3,2'-, 2,2'-, 3,3'-, and 3,4'-BTPs (Figure 2).

**Scheme 2:**
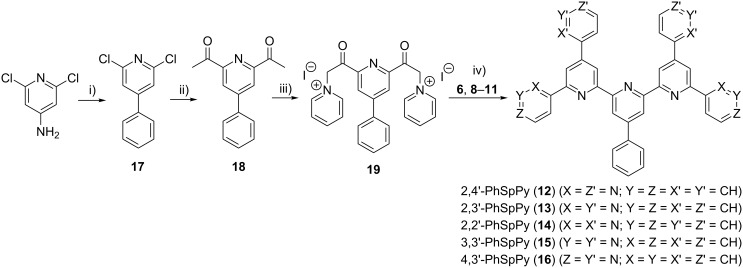
Synthetic pathway to PhSpPys (**12**–**16**). i) Cu_2_O, isoamylnitrite, benzene, 100 °C, 3 h; ii) (1-ethoxy)-vinyl-tributylstannane, Pd(PPh_3_)_4_, DMF, 20 h reflux, acetone, HCl; iii) iodine, pyridine, 100 °C, 4 h; iv) NH_4_OAc, MeOH reflux.

The synthesis of these phenylseptipyridines, which follows the same reaction scheme as for the BTPs, is presented in Scheme 2. The required phenylpyridine bis(pyridinium iodine) salt is synthesized analogously to the corresponding pyrimidine compound. In this way, five new PhSpPys (2,4'-, 2,3'-, 2,2'-, 3,3'-, and 4,3'-PhSpPy) (**12**–**16**) were be obtained in mediocre yield but with high purity.

### STM and calculations

**Figure 3:**
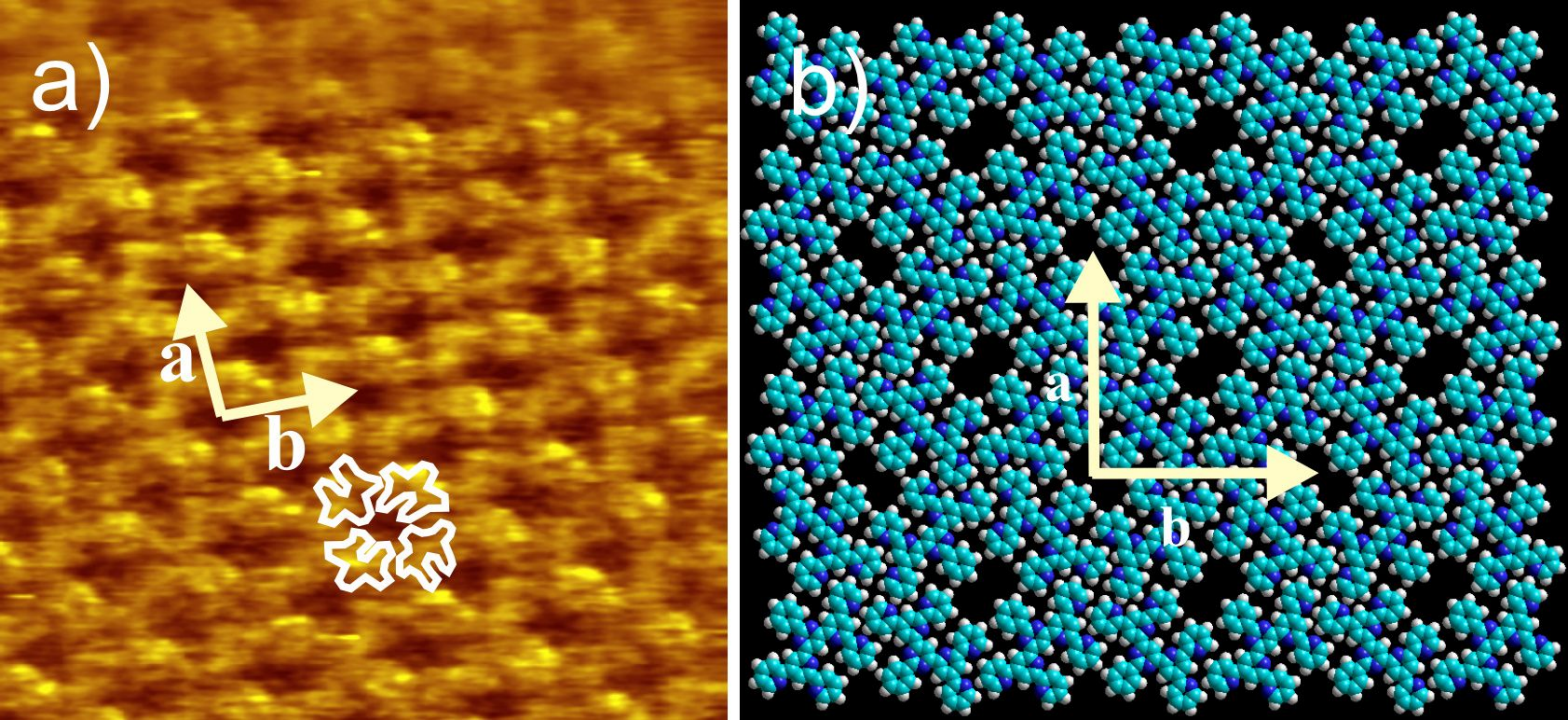
a) 15 × 15 nm^2^ STM image (*I*_set_ = 14 pA, *V*_set_ = −0.64 V) of 2,2'-BTP (**5**) at the HOPG/TCB interface [*a* = 3.0 ± 0.2 nm, *b* = 3.0 ± 0.2 nm, 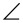
*_a,b_* = 91 ± 2°] with four overlaid molecules; b) model of the 2,2'-BTP (**5**) square pattern.

**Figure 4:**
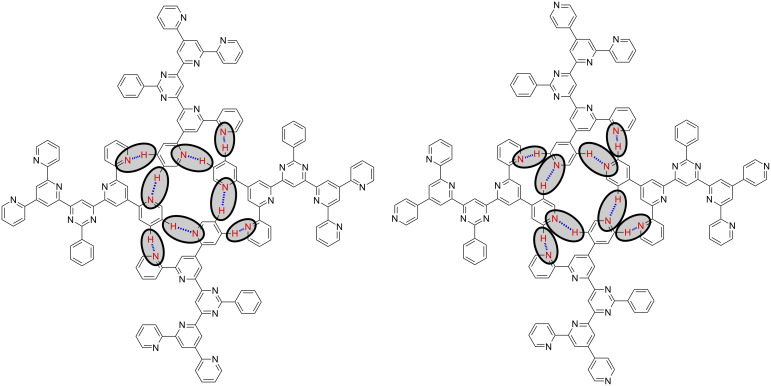
Hydrogen bonding motif of 2,2'-BTP (**5**) (left) and of 2,4'-BTP (**2**) [6–7] (right) found experimentally; the hydrogen bonds are marked by ovals.

The self-assembly properties of the newly-synthesized oligopyridines in 1,2,4-trichlorobenzene (TCB) solution were investigated at the HOPG/liquid interface and compared to the already known BTPs. The 2D structure of 2,2'-BTP (**5**) can be seen in Figure 3. A square pattern of dark spots surrounded by bright areas with a unit cell of *a* = 3.0 ± 0.2 nm, *b* = 3.0 ± 0.2 nm, and an angle 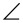
*_a,b_* = 91 ± 2° is observed. A closer look at the bright areas reveals small bright spots, which we attribute to the single (hetero)aromatic rings. The submolecular resolution allows for the suggestion of a tentative model. Self-assembly can be explained by the presence of intermolecular hydrogen bonds, which is already known from the previous BTPs [7]. Interestingly, comparison of the unit cell of self-assembled 2,2'-BTP (**5**) with the unit cell of 2,4'-BTP (**2**) shows almost identical data (*a* = *b* = 3.1 nm ± 0.2, 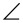
*_a,b_* = 90 ± 1°) [6–7]. The structural agreement can be understood by taking a closer look at the molecular structures of both compounds. Here, it can be seen that the exchange of the *para*-connected pyridine ring in 2,4'-BTP (**2**) by the *ortho*-connection in 2,2'-BTP (**5**) maintains the hydrogen bonding pattern and thus leads to the corresponding 2D structure (Figure 4). No further polymorph could be found for 2,2'-BTP (**5**) at the HOPG/TCB solution interface.

**Figure 5:**
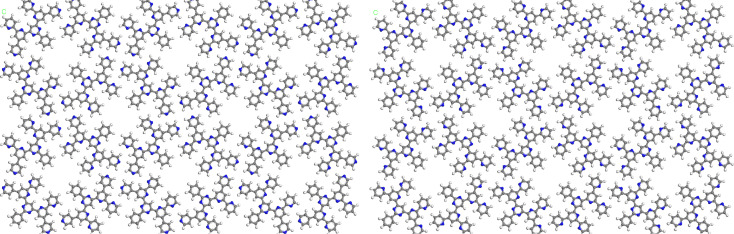
Adsorbate structures of 2,4'-BTP (**2**) (left) and 2,2'-BTP (**5**) (right) in the square symmetric structure, optimized with the Compass [32] force field.

To further support the experimental results, force field calculations of the square symmetric adsorbate layers of 2,4'-BTP (**2**) and 2,2'-BTP (**5**) were performed. The results of these calculations underline their structural similarities: For both isomers, the BTP molecules arrange in a similar fashion (Figure 5), forming intermolecular hydrogen bonds at comparable positions. In addition, the lattice constants of the relaxed network differ by less than 1%. For 2,4'-BTP (**2**), the Compass-optimized [32] lattice constant is 3.23 nm, and it is 3.26 nm for 2,2'-BTP (**5**).

Despite the similarity of the calculated structures, small differences were found in the energies of the hydrogen bonds. In the 2,4'-BTP (**2**) network, hydrogen bonds of −37.2 kJ mol^−1^ per molecule in the unit cell were obtained from the Compass [32] optimization. For 2,2'-BTP, a slightly weaker intermolecular bonding of −23.9 kJ mol^−1^ per molecule was calculated.

As the self-assembly of the oligopyridines is dominated by the orientation of the peripheral nitrogen atoms, 2,2'- (**14**) and 3,3'-PhSpPy (**15**) should display the 2D structures corresponding to 2,2'- (**5**) and 3,3'-BTP (**3**), respectively.

**Figure 6:**
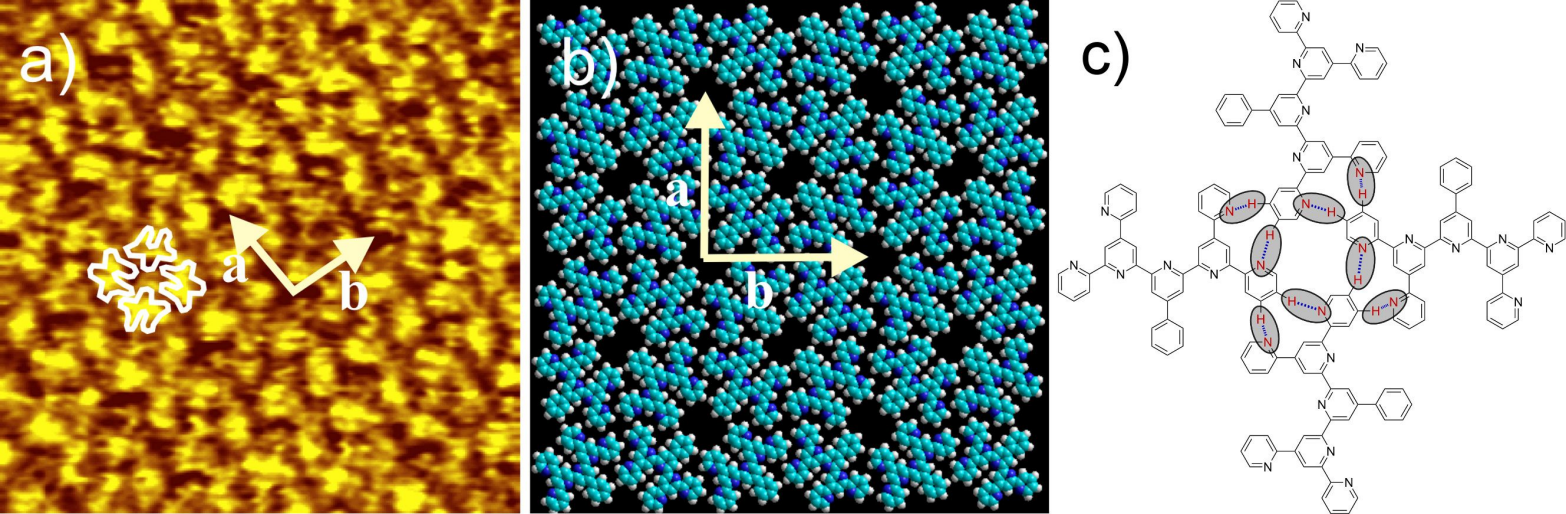
a) 15 × 15 nm^2^ STM image (*I*_set_ = 3.41 nA, *V*_set_ = −660 mV) of 2,2'-PhSpPy (**14**) at the HOPG/TCB interface [*a* = 3.0 ± 0.1 nm, *b* = 3.0 ± 0.1 nm, 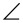
*_a,b_* = 92 ± 3°] with four overlaid molecules; b) model of the 2,2'-PhSpPy (**14**) square pattern; c) hydrogen bonding motif of 2,2'-PhSpPy (**14**); the hydrogen bonds are marked by ovals.

Although the resolution of the STM image is significantly lower than for 2,2'-BTP (**5**) (Figure 3) a square symmetric structure for 2,2'-PhSpPy (**14**) at the HOPG/TCB solution interface can be detected (Figure 6), just as for 2,2'-BTP (**5**). The unit cell dimensions (*a* = 3.0 ± 0.1 nm, *b* = 3.0 ± 0.1 nm, 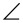
*_a,b_* = 92 ± 3°) are essentially identical to those of the 2D structures of 2,2'-BTP (**5**) and 2,4'-BTP (**2**) (see above). Based on these observations, it is likely that a corresponding intermolecular hydrogen bonding pattern gives rise to the square packing motif (Figure 6).

**Figure 7:**
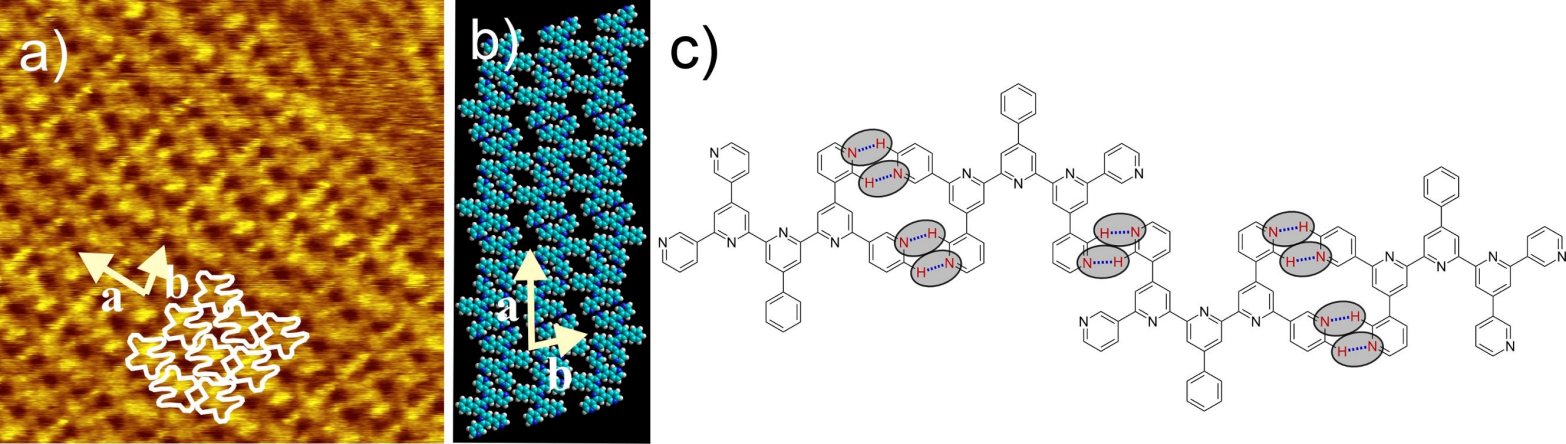
a) 15 × 15 nm^2^ STM image (*I*_set_ = 14.5 pA, *V*_set_ = −610 mV) of 3,3'-PhSpPy (**15**) at the HOPG/TCB interface [*a* = 2.7 ± 0.2 nm, *b* = 1.6 ± 0.2 nm, 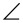
*_a,b_* = 77 ± 2°] overlaid with nine molecules; b) model of the 3,3'-PhSpPy (**15**) lamellar pattern; c) hydrogen bonding motif of 3,3'-PhSpPy (**15**); the hydrogen bonds are marked by ovals.

3,3'-BTP (**3**) is known to show four different 2D structures at the HOPG/TCB solution interface depending on the concentration: Three linear packing patterns and one hexagonal pattern [7,9]. For 3,3'-PhSpPy (**15**) only one 2D structure could be found with no dependence on the concentration, that is, a lamellar pattern with unit cell parameters of *a* = 2.7 ± 0.2 nm, *b* = 1.6 ± 0.2 nm, 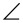
*_a,b_* = 77 ± 2° (Figure 7 and Figure S1). Those parameters correspond well to the data for a linear polymorph of 3,3'-BTP (**3**) (LinI, *a* = 2.9 ± 0.2 nm, *b* = 1.6 ± 0.2 nm, 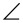
*_a,b_* = 78 ± 1°) [7]. Interestingly, for 3,3'-BTP (**3**) the most densely packed structure was much more abundant than LinI, whereas for 3,3'-PhSpPy (**15**) the corresponding densely packed 2D assembly could not be found.

**Figure 8:**
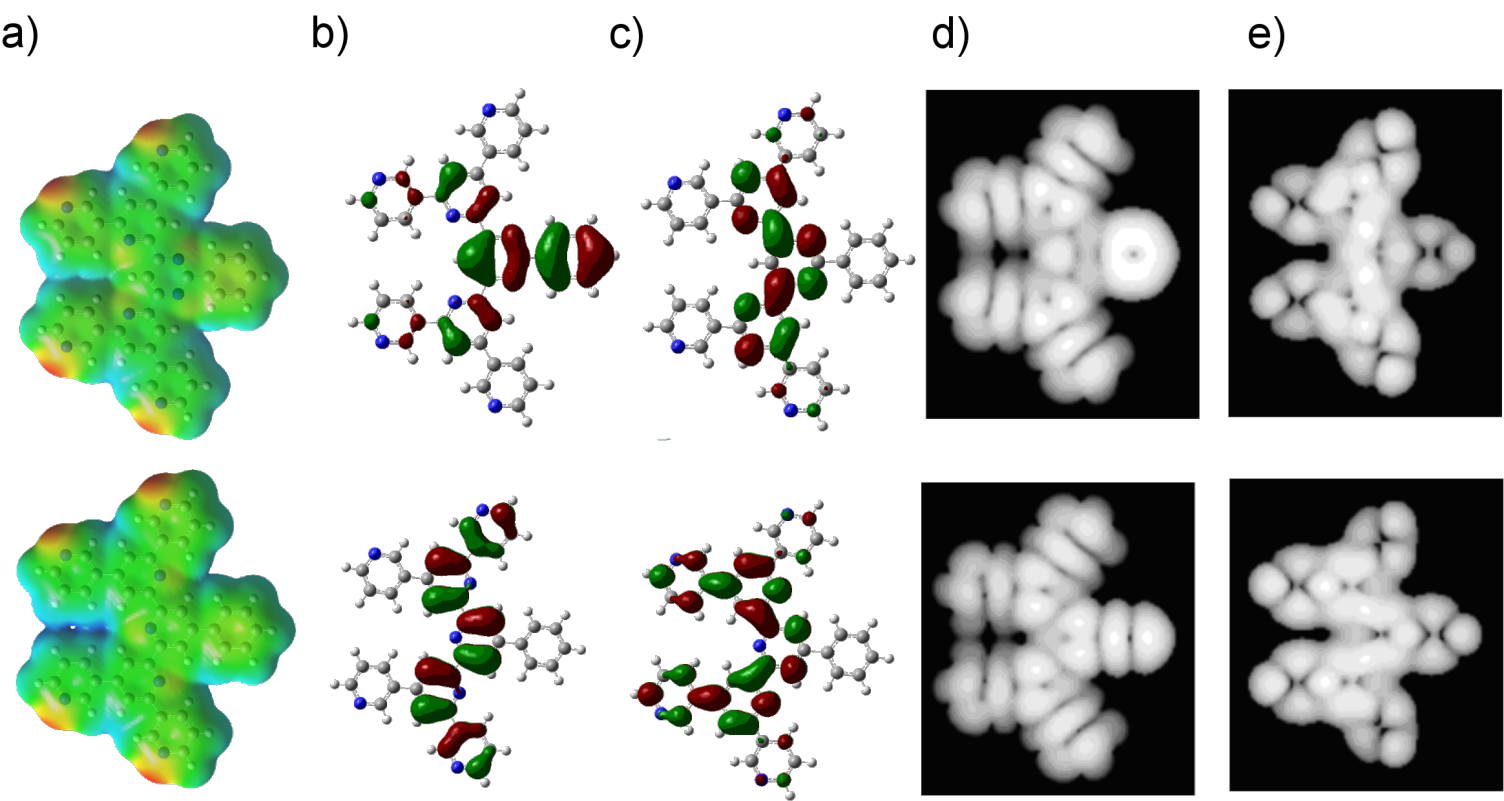
Electronic properties for 3,3'-BTP (**3**) (top) and 3,3'-PhSpPy (**15**) (bottom). (a) Electrostatic potential, (b) HOMO, (c) LUMO, and simulated STM images of (d) occupied and (e) unoccupied orbitals. The energy levels of LUMO and HOMO for 3,3'-BTP (**3**) are −9.46 kJ mol^−1^ and −24.31 kJ mol^−1^ , respectively, and for 3,3'-PhSpPy (**15**) −8.59 kJ mol^−1^ and −23.83 kJ mol^−1^, respectively.

With density functional theory calculations, we can show that BTPs and corresponding PhSpPys both possess similar electronic structure. As an example, Figure 8 shows electronic properties for 3,3'-BTP (**3**) and the corresponding 3,3'-PhSpPy (**15**).

The electrostatic potential maps indeed illustrate the similarity of the two different oligopyridines. In both cases, an accumulation of negative charge on the peripheral nitrogen atoms can be observed, whereas the rest of the molecule shows no charge accumulation. While the molecules are very similar, a closer look at the frontier orbitals reveals key differences. In both isomers, the HOMO and LUMO are mainly localized on the backbone pyridine and the pyridines directly connected to it. In the 3,3'-PhSpPy (**15**) molecule, the HOMO extends to the backbone phenyl group, whereas in 3,3'-BTP (**3**) one of the peripheral pyridyl groups contributes to the HOMO. Only for 3,3'-BTP (**15**) an extension of the LUMO to the peripheral pyridines was observed.

The HOMO level is at an energy of −24.31 kJ mol^−1^ for 3,3'-BTP (**3**) and −23.83 kJ mol^−1^ for 3,3'-PhSpPy (**15**). The LUMO levels are at −9.46 kJ mol^−1^ and −8.59 kJ mol^−1^ for 3,3'-BTP (**3**) and 3,3'-PhSpPy (**15**), respectively. Still, it has to be noted that the accuracy of density functional theory for unoccupied states and HOMO–LUMO gaps is rather limited.

Even though there are slight differences in the simulated HOMO and LUMO orbitals, several calculated orbitals must be overlaid for comparison with experimental STM images [33]. With a finite range of orbitals contributing to an STM image, less importance is attributed to the differences in the single orbitals. As Figure 8 shows, the simulated STM images for 3,3'-BTP (**3**) and 3,3'-PhSpPy (**15**) are very similar: Only the phenyl ring, which is not affected by the intermolecular hydrogen bonding, shows any difference.

The similarity of the formed 2D structures of both the BTPs and the corresponding PhSpPys supports the hypothesis that the position of the nitrogen atoms in the central aromatic moiety plays only a minor role for pattern formation, and that the orientation of the peripheral pyridine nitrogen atoms is critical. Thus, we assume that the structures that are formed by the remaining three PhSpPys, with different peripheral pyridine ring orientations, can be seen as substitutes for the structures of the missing BTPs.

All three PhSpPys show lamellar 2D structures at the HOPG/TCB solution interface and only one assembly could be found for each compound.

**Figure 9:**
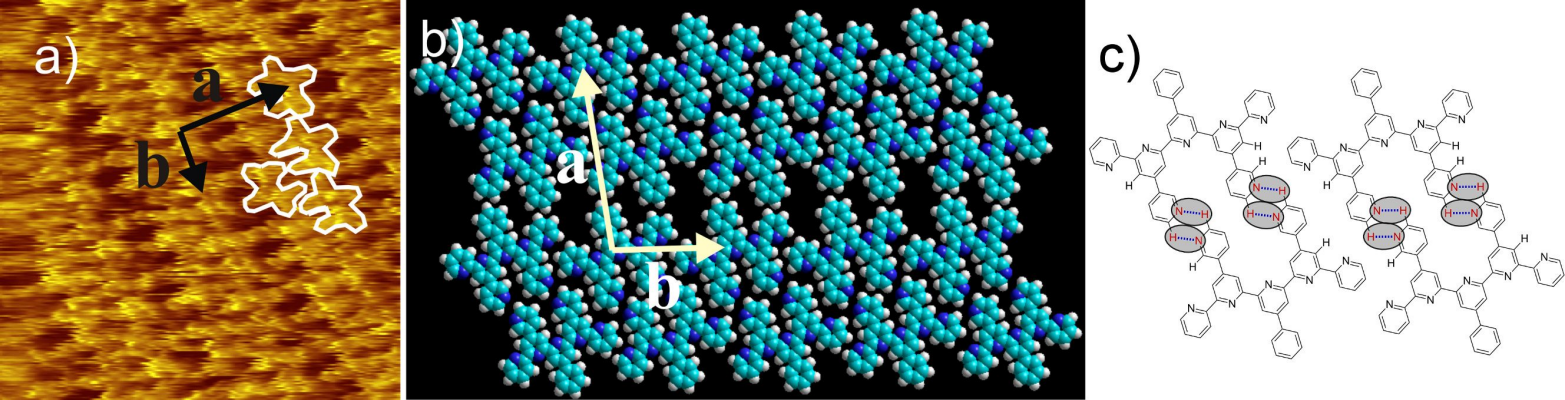
a) 16 × 16 nm^2^ STM image (*I*_set_ = 2.1 nA, *V*_set_ = −0.60 V) of 2,3'-PhSpPy (**13**) at the HOPG/TCB interface [*a* = 2.8 ± 0.1 nm, *b* = 1.5 ± 0.1 nm, 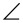
*_a,b_* = 93 ± 2°] with four overlaid molecules; b) model of the 2,3'-PhSpPy (**13**) lamellar pattern; c) hydrogen bonding motif of 2,3'-PhSpPy (**13**); the hydrogen bonds are marked by ovals.

2,3'-PhSpPy (**13**) self-assembles into an almost rectangular structure (*a* = 2.8 ± 0.1 nm, *b* = 1.5 ± 0.1 nm, 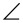
*_a,b_* = 93 ± 2°), where the bright lamellae are clearly separated by slightly elongated dark spots (Figure 9 and Figure S1). In the bright areas, a substructure can be observed, which leads to a tentative model with pairs of PhSpPy molecules pointing with their “legs” to each other and assembled in rows (lamellae). There are intra- and interpair interactions based on single and double intermolecular hydrogen bonds between neighboring *ortho*-connected pyridine rings. There are no hydrogen bonding interactions between the rows. A further conformational arrangement could be imagined where the peripheral 3-pyridyl units point with both N-atoms in the opposite direction at the expense of attracting C–H^…^N interactions and forming a repulsive N^...^N interaction. An estimation of the interactions based on C–H^…^N double bond and single bond dimers [7] yields −36.5 kJ mol^−1^ for the conformation shown in Figure 9 and −29.5 kJ mol^−1^ for the alternative conformation, thus clearly favoring the first one.

**Figure 10:**
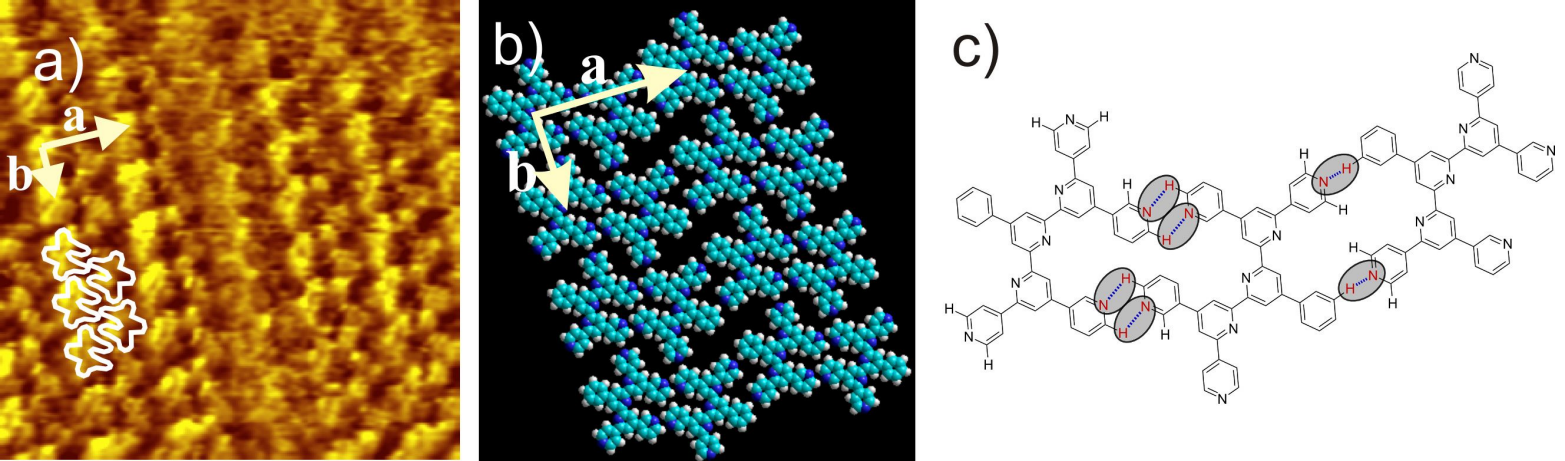
a) 15 × 15 nm^2^ STM image (*I*_set_ = 2.51 nA, *V*_set_ = −600 mV) of 4,3'-PhSpPy (**16**) at the HOPG/TCB interface [*a* = 2.8 ± 0.2 nm, *b* = 1.7 ± 0.2 nm, 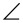
*_a,b_* = 93 ± 3°] with five overlaid molecules; b) model of the 4,3'-PhSpPy (**16**) lamellar pattern; c) hydrogen bonding motif of 4,3'-PhSpPy (**16**); the hydrogen bonds are marked by ovals.

A similar 2D structure is found for 4,3'-PhSpPy (**16**) displaying a unit cell with *a* = 2.8 ± 0.2 nm, *b* = 1.7 ± 0.2 nm, and 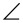
*_a,b_* = 93 ± 3° (Figure 10). We suggest a model with the corresponding intermolecular interactions as for 2,3'-PhSpPy (**13**), i.e., the *ortho*-connected pyridine rings seem to dominate the packing pattern. An energetic estimation, corresponding to the considerations above, makes an alternative conformation with both nitrogen atoms of the 3-pyridyl rings pointing in the opposite direction unlikely.

**Figure 11:**
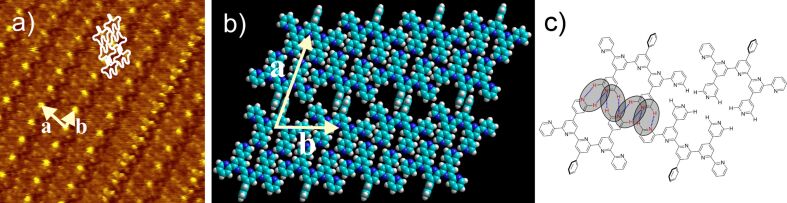
a) 15 × 15 nm^2^ STM image (*I*_set_ = 23.5 pA, *V*_set_ = −580 mV) of 2,4'-PhSpPy (**12**) at the HOPG/TCB interface [*a* = 2.4 ± 0.2 nm, *b* = 1.5 ± 0.2 nm, 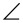
*_a,b_* = 77 ± 2°] with six overlaid molecules; b) model of the 2,4'-PhSpPy (**12**) lamellar pattern; c) hydrogen bonding motif of 2,4'-PhSpPy (**12**); the hydrogen bonds are marked by ovals.

2,4'-PhSpPy (**12**) displays a more dense 2D structure than the previous oligopyridines expressed by rows of bright spots leading to a unit cell with *a* = 2.4 ± 0.2 nm, *b* = 1.5 ± 0.2 nm, and 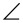
*_a,b_* = 77 ± 2° (Figure 11). The resolution of the STM image does not allow a direct assignment of the molecules to the contrast. Thus, we suggest a tentative model, which shows reasonable hydrogen bonding interactions primarily between the peripheral *para*-connected pyridine units. The model implies dense packing, such that the phenyl rings cannot be coplanar. Rather, they must stand upright, stabilizing the packing by van der Waals interactions. This arrangement might be the reason for the relatively high electron density which is seen as the bright spots in Figure 11a.

## Conclusion

The series of nine possible isomeric bis(terpyridine)-derived oligopyridines (BTPs) was extended by one further member, 2,2'-BTP (**5**). The still missing four isomers were synthetically not accessible, so the class of phenylseptipyridines (PhSpPys) was introduced, which differ from the BTPs only in the central aromatic moiety. The hypothesis that the backbone system of the oligopyridine does not affect 2D self-assembly at the HOPG/solution interface if the orientation of the peripheral nitrogen atoms in the BTPs and the PhSpPys stays the same, was supported by comparing the STM images of the pairs 2,2'-BTP (**5**)/2,2'-PhSpPy (**14**) and 3,3'-BTP (**3**)/3,3'-PhSpPy (**15**). DFT calculations carried out for additional support showed that the electronic structures of each PhSpPy and its corresponding BTP are similar, so a greatly differing behavior is not expected. Although only a single 2D structure was found at the HOPG/liquid interface for each of the new compounds we cannot exclude the possibility of further (pseudo)polymorphs such as is the case for the BTPs, especially for 3,3'-BTP [7,9]. Further experiments with varying concentration and/or different solvents are required to elucidate this issue.

Three new peripheral orientations of the nitrogen atoms in the pyridine rings were obtained using the PhSpPy isomers: 2,3'-PhSpPy (**13**), 3,3'-PhSpPy (**15**), and 4,3'-PhSpPy (**16**). These compounds correspond to three unavailable BTPs because of the inaccessibility of the corresponding three diazachalcone precursors. All of the resulting 2D structures showed lamellar patterns, which are stabilized by the expected hydrogen bonding interactions. Thus, the presented substitution strategy successfully allowed for the near completion of the series, with just one isomer (4,4'-BTP) missing. Such 2D assemblies broaden the understanding of structure formation toward 2D crystal engineering: Additionally, they may act as templates for the creation of hybrid nanostructures, which are under investigation.

## Experimental

### Scanning tunneling microscopy

STM measurements were carried out under ambient conditions with a low-current RHK 1000 control system. Before the desired measurements the tip was tested by imaging the HOPG surface. These HOPG measurements were used for internal calibration. Then 2 µL solutions of the respective oligopyridine with a concentration of 0.04 mg mL^−1^ (4,3'-PhSpPy (**16**)), 0.05 mg mL^−1^ (2,3'-PhSpPy (**13**) and 3,3'-PhSpPy (**15**)), and 0.20 mg mL^−1^ (2,2'-BTP (**5**), 2,2'-PhSpPy (**14**), and 2,4'-PhSpPy (**12**)) in TCB were prepared. All images presented were obtained in constant current mode using a Pt/Ir (90/10) tip, which was mechanically sharpened. The bias was applied to the tip. The raw STM images were smoothed and the heights were compensated with the program XPMPro2.0.0.8™ (RHK). Some images were Fourier transformed and filtered. The errors given for the unit cells were determined by averaging over several unit cells. The models for the monolayers are constructed with the help of the program Hyperchem (version 7.01, 2002, Hypercube, Inc.) and implemented MM+ force field. The single molecules were positioned manually in van der Waals contact with van der Waals radii for the H–H contacts of 82.5 pm, as given by the program.

### Calculations

Gaussian 03 [34] density functional theory calculations were carried out with the B3YLP/6-311G [35–36] method. Electrostatic potentials were mapped onto a constant electron density surface. For the simulated STM images, a simple Tersoff–Hamann [37] approach was used. Orbitals in a given energy range close to the frontier orbitals were added and the resulting density was plotted. The orbitals were generated with Gaussian 03 from geometries that were obtained from relaxation of planar systems. For the addition and visualization steps, a homemade python script was used. Force field results were obtained from geometry optimizations of oligopyridine layers carried out with the Compass [32] force field as implemented in the Accelrys Materials Studio program package.

## Supporting Information

Supporting information describes the synthesis, purification and characterization data of all substances given in this article and some magnified STM images of selected compounds.

File 1 Experimental details.
