# Supplementary material for: Septipyridines as conformationally controlled substitutes for inaccessible bis(terpyridine)-derived oligopyridines in two-dimensional self-assembly
Source: Beilstein J Nanotechnol. 2011 Jul 26;2:405–15. doi: 10.3762/bjnano.2.46 (PMC3190612; doi:10.3762/bjnano.2.46)
Supplement: File 1 — Experimental details. [file Beilstein_J_Nanotechnol-02-405-s001.pdf]

Supporting Information

for

Septipyridines as conformationally controlled substitutes for inaccessible bis(terpyridine)-derived oligopyridines in two-dimensional self-assembly

Daniel Caterbow[1], Daniela Künzel[2], Michael G. Mavros[1], Axel Groß[2], Katharina Landfester[3] and Ulrich Ziener[*1]

Address: [1]Institute of Organic Chemistry III/Macromolecular Chemistry, [2]Institute of Theoretical Chemistry, University of Ulm, Albert-Einstein-Allee 11, D-89081 Ulm, Germany and [3]Max Planck Institute for Polymer Research, Ackermannweg 10, D-55128 Mainz, Germany

Germany

Email: Ulrich Ziener* - ulrich.ziener@uni-ulm.de

*Corresponding author

Experimental details

Synthesis

General

Phenylpyrimidine bis(pyridinium iodine) salt **7** [1], the diazachalcones **6,** and **8-11** [2,3] and tributyl(1-ethoxyvinyl)stannane [4] were prepared according to the literature. 2-Pyridinecarbaldehyde (98%+), iodine (100%), Na_2CO_3 (99%), and methanol (99.9%+) from Merck, NH_4OAc (99%) from VWR, copper(I) oxide (97%) and $MgSO_4$ (97%) from Acros, isoamylnitrite (97%) and 2,6-dichloro-4-aminopyridine (97%) from Alfa Aesar, $Pd(PPh_3)_4$ (99.9%) from Strem, 2-acetylpyridine (98%+) and KF (99%) from Fluka, and 1,2,4-trichlorobenzene (TCB) (+99%) from Aldrich were used as received. When water-free solvents were employed, they were dried according to the literature [5]. The other reagents and solvents were used without further purification. The NMR data were obtained on a Bruker DRX 400 and DRX 500 spectrometer, calibrated against the solvent signal ($CDCl_3$: ^{1}H NMR: δ = 7.27; ^{13}C NMR: δ = 77.0; [D6]DMSO: ^{1}H NMR: δ = 2.50; [D2]1,1,2,2-tetrachloroethane: ^{1}H NMR: δ = 6.00; ^{13}C NMR: δ = 74.2) and are given in ppm. The assignment of the ^{1}H NMR signals is made by comparison with similar compounds described in [1,6], increment calculations, and simulations of the spectra with ACD/HNMR Predictor, Version 3.00, 1997.

2,2'-BTP (5).

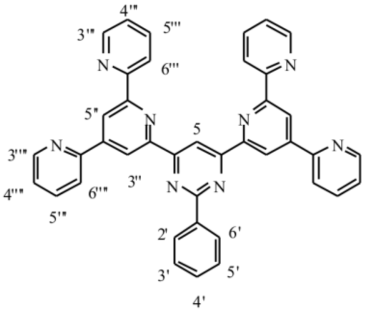

2-Acetylpyridine (3.53 g, 33.0 mmol) and 2-pyridinecarbaldehyde (4.00 g, 33.0 mmol) were added dropwise to a 25 wt % Na_2CO_3 solution (100 mL) and stirred for 1 h at 70 °C. After cooling down the solid precipitate was filtered off and dried under vacuum at rt. The resulting chalcone **6** was obtained as a brown solid (6.03 g) and used for the next step without further purification. NH_4OAc (2.26 g, 29.3 mmol), (1) (259 mg, 1.23 mmol) and phenylpyrimidine bis(pyridinium iodine) salt **7** (400 mg, 0.615 mmol) were suspended in methanol (20 mL) and heated under reflux for 30 min. A beige solid of 2,2'-BTP (**5**) precipitated in the heat and was centrifuged and dried under vacuum. Yield: 62.0% (236 mg, 0.382 mmol). ^{1}H NMR (500 MHz, 100 °C, TCE-d_2): δ 9.81 (1H, s, H^5), 9.37 (2H, d, ^4J = 1.5 Hz, $H^{3''}$ or $H^{5''}$), 9.27 (2H, d, ^4J = 1.5 Hz, $H^{3''}$ or $H^{5''}$), 8.94–8.84 (8H, m, $H^{2'}$, $H^{6'}$, $H^{3'''}$, $H^{6'''}$ and $H^{3''''}$), 8.15 (2H,

dt, ^3J = 8.0 Hz, ^4J = 1.0 Hz, $H^{6''''}$), 7.99–7.94 (4H, m, $H^{5'''}$ and $H^{5''''}$), 7.69–7.65 (2H, m, $H^{3'}$ and $H^{5'}$), 7.65–7.60 (1H, m, $H^{4'}$), 7.47–7.44 (4H, m, $H^{4'''}$ and $H^{4''''}$); ^{13}C NMR (500 MHz, 100 °C, TCE-d2): δ 164.7, 157.0, 156.4, 155.4, 155.2, 150.4, 149.5, 149.3, 138.5, 137.4, 136.8, 138.9, 129.0, 128.8, 124.2, 124.0, 121.7, 121.6, 120.6, 119.6, 112.6, 99.9 MALDI-TOF: calculated *m*/*z* for $C_{40}H_{26}N_8$: $[M + H]^+$ 619.67, found 619. 97; elemental analysis: calculated: %C 77.65, %H 4.24, %N 18.11; found: %C 77.25, %H 4.31, %N 17.99.

2,6-Dichloro-4-phenylpyridine (17).

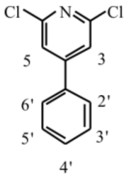

To 2,6-dichloro-4-aminopyridine (3.53 g, 21.7 mmol) and copper(I) oxide (3.89 g, 27.2 mmol), isoamylnitrite (11 mL) and dry benzene (150 mL) were added under Ar. The mixture was refluxed for 6 h. After cooling to rt, a solid was formed and filtered off. From the residual filtrate the solvent was removed. The residue was dissolved in $CHCl_3$/hexane (1:1, v:v) and purified by column chromatography. ($CHCl_3$/hexane (1:1, v:v), alumina, R_f = 0.8) leading to **17** as a white solid. Yield: 30.2% (1.47 g, 6.58 mmol). ^{1}H NMR (400 MHz, $CDCl_3$): δ 7.61–7.58 (2H, m, $H^{2'}$ and $H^{6'}$), 7.53–7.50 (3H, m, $H^{3'}$, $H^{4'}$ and $H^{5'}$), 7.47 (2H, s, H^3 and H^5); ^{13}C NMR (400 MHz, $CDCl_3$): δ 153.9, 151.0, 135.7, 130.2, 129.4, 127.0, 120.8.

2,6-Diacetyl-4-phenylpyridine (18).

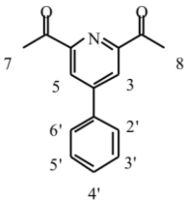

2,6-Dichloro-4-phenylpyridine **(17)** (1.48 g, 6.60 mmol), tributyl(1-ethoxyvinyl)stannane (5.29 g, 14.6 mmol), and $Pd(PPh_3)_4$ (0.354 mg, 0.306 mmol) in dry DMF (50 mL) were refluxed for 20 h under Ar. After cooling to rt the reaction mixture was poured onto a mixture of KF (1.31 g, 22.6 mmol), distilled water (60 mL) and ether (60 mL) and stirred for 15 min. A grey solid was filtered off and slightly washed with ether. The filtrate was extracted three times with ether; the organic phases were collected and dried over $MgSO_4$. After removing the solvent the bis(enol ether) remained as a brown solid. The solid was dissolved in acetone and a few drops of HCl (1M) were added. After stirring for 24 h at rt and removing the solvent, $CHCl_3$ and a saturated solution of $NaHCO_3$ was added. The product was extracted

several times with $CHCl_3$ and dried over $MgSO_4$. Removing the solvent delivered **18** as a beige-brown solid, which was recrystallized from methanol and dried under vacuum. Yield: 44.0% (694 mg, 2.90 mmol). ^{1}H NMR (400 MHz, $CDCl_3$): δ 8.47 (2H, s, H^{3} and H^{5}), 7.74–7.70 (2H, m, $H^{2'}$ and $H^{6'}$), 7.49–7.56 (3H, m, $H^{3'}$, $H^{4'}$ and $H^{5'}$), 2.84 (6H, s, H^{7} and H^{8}); ^{13}C NMR (100 MHz, $CDCl_3$): δ 199.7, 153.6, 150.8, 137.0, 130.1, 129.5, 127.3, 122.5, 25.9; MS (CI) *m*/*z* calculated for $C_{15}H_{13}NO_2$: $[M + H]^+$: 240.06 $[M + H]^+$, found: 240.06; elemental analysis: calculated: %C 73.30, %H 5.48, %N 5.85; found: %C 73.66, %H 5.33, %N 5.84.

Phenylpyridine bis(pyridinium iodine) salt 19.

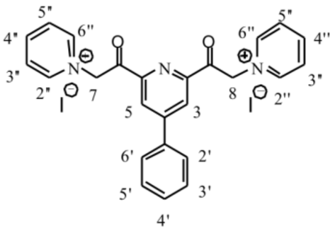

2,6-Diacetyl-4-phenylpyridine **(18)** (398 mg, 1.66 mmol) and iodine (942 mg, 3.71 mmol) in dry pyridine (7 mL) were refluxed under Ar for 4 h. After cooling to rt the product **19** as a beige solid was filtered off, cautiously washed with methanol, and dried under vacuum. Yield: 55.2% (478 mg, 0.916 mmol). ^{1}H NMR (400 MHz, DMSO-d_6): δ 9.13 (4H, d, ^{3}J =5.6 Hz, $H^{2''}$ and $H^{6''}$) 8.80 (2H, t, ^{3}J = 4.0 Hz, $H^{4''}$), 8.64 (2H, s, H^{3} and H^{5}), 8.35 (4H, t, ^{3}J = 3.2 Hz, $H^{3''}$ and $H^{5''}$), 8.02–7.99 (2H, m, $H^{2'}$ and $H^{6'}$), 7.65–7.60 (3H, m, $H^{3'}$, $H^{4'}$ and $H^{5'}$), 6.72 (4H, s, H^{7} and H^{8}); MS (CI) *m*/*z* calculated for $C_{25}H_{21}I_2N_3O_2$: 240.27 $[M\text{-}2I\text{-}2pyridine + H]^+$, found: 240.17; elemental analysis: calculated: %C 46.25, %H 3.26, %N :6.47; found: %C 45.93, %H 3.62, %N 6.77.

2,2'-PhSpPy (14).

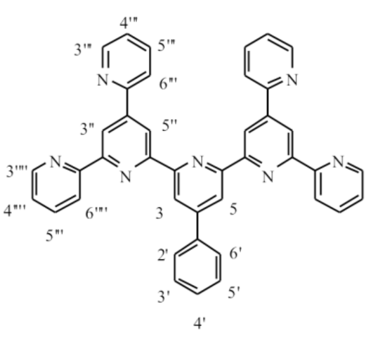

2,2'-Azachalcone **6** (65.2 mg, 0.310 mmol), phenylpyridine bis(pyridinium iodine) salt **19** (100 mg, 0.154 mmol) and NH_4OAc (700 mg, 9.09 mmol) were refluxed in methanol (6 mL) for 24 h. **14** precipitated as an off-white solid. It was filtered off, washed with methanol, and dried under vacuum. Yield: 26.1% (24.8 mg, 0.0402 mmol). ^{1}H NMR (500 MHz, 100 °C,

TCE-d_2): δ 9.46 (2H, d, ^4J = 1.5 Hz, $H^{3''}$ or $H^{5''}$), 9.18 (2H, d, ^4J = 1.5 Hz, $H^{3''}$ or $H^{5''}$), 9.00 (2H, s, H^3 and H^5), 8.92–8.90 (2H, m, $H^{3'''}$, $H^{3''''}$ or $H^{6''''}$), 8.84–8.82 (2H, m, $H^{3'''}$, $H^{3''''}$ or $H^{6''''}$), 8.78–8.75 (2H, m, $H^{3'''}$, $H^{3''''}$ or $H^{6''''}$), 8.19 (2H, d, ^3J = 8.0 Hz, $H^{6'''}$), 8.02 (2H, d, ^3J = 8.0 Hz, ($H^{2'}$ and $H^{6'}$) or ($H^{3'}$ und $H^{5'}$)), 7.97–7.92 (4H, m, $H^{5'''}$ and $H^{5''''}$), 7.67 (2H, t, ^3J = 7.5 Hz, ($H^{2'}$ and $H^{6'}$) or ($H^{3'}$ and $H^{5'}$)), 7.59 (1H, t, ^3J = 7.5 Hz, $H^{4'}$), 7.47–7.40 (4H, m, $H^{4'''}$ and $H^{4''''}$); MALDI-TOF: calculated *m/z* for $C_{41}H_{27}N_7$: 619.66 $[M + 2H]^+$, found: 619.48; elemental analysis: calculated: %C 79.72, %H 4.41, %N 15.87; found: %C 79.48, %H 4.48, %N: 15.67.

2,3'-PhSpPy (13).

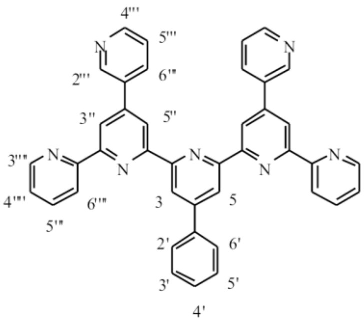

2,3'-Diazachalcone **9** (53.8 mg, 0.256 mmol), phenylpyridine bis(pyridinium iodine) salt **19** (76.0 mg, 0.117 mmol), and NH_4OAc (550 mg, 7.14 mmol) were refluxed in methanol (7 mL) for 24 h. **13** precipitated as an off-white solid. It was filtered off, washed with methanol and dried under vacuum. Yield: 15.9% (11.5 mg, 0.0186 mmol). ^1H NMR (500 MHz, 100 °C, TCE-d_2): δ 9.21–9.20 (2H, m, $H^{3''}$, $H^{5''}$, or $H^{2'''}$), 9.02 (2H, s, H^3 and H^5), 9.00 (2H, d, ^4J = 1.5 Hz, $H^{3''}$, $H^{5''}$, or $H^{2'''}$), 8.86 (2H, d, ^4J = 2.0 Hz, $H^{3''}$, $H^{5''}$, or $H^{2'''}$), 8.82–8.80 (2H, m, $H^{3''''}$ or $H^{6''''}$), 8.79 (2H, dd, ^3J = 3.5 Hz, ^3J = 5.0 Hz, $H^{3''''}$ or $H^{6''''}$), 8.76 (2H, d, ^3J = 8.0 Hz, $H^{4'''}$), 8.24 (2H, dt, ^4J = 2.0 Hz, ^3J = 5.0 Hz, $H^{6'''}$), 8.01 (2H, d, ^3J = 8.0 Hz, ($H^{2'}$ and $H^{6'}$) or ($H^{3'}$ and $H^{5'}$)), 7.95 (2H, td, ^4J = 1.5 Hz, ^3J = 7.5 Hz, $H^{5''''}$), 7.68 (2H, t, ^3J = 7.5 Hz, ($H^{2'}$ and $H^{6'}$) or ($H^{3'}$ and $H^{5'}$)), 7.61–7.58 (1H, m, $H^{4'}$), 7.53 (2H, dd, ^3J = 5.0 Hz, ^3J = 8.0 Hz, $H^{5'''}$), 7.42 (2H, ddd, ^4J = 1.0 Hz, ^3J = 5.0 Hz, ^3J = 7.5 Hz, $H^{4''''}$); MALDI-TOF: calculated *m/z* for $C_{41}H_{27}N_7$: 619.66 $[M + 2H]^+$, found: 619.63; elemental analysis: calculated: %C 79.72, %H 4.41, %N 15.87; found: %C 79.46, %H 4.53, %N 15.90.

2,4’-PhSpPy (12).

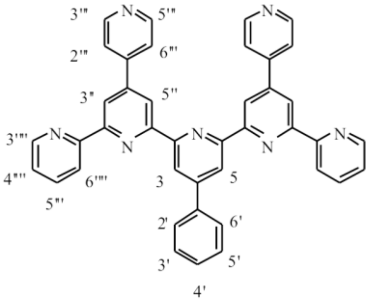

2,4'-Diazachalcone **10** (65.2 mg, 0.310 mmol), phenylpyridine bis(pyridinium iodine) salt **19** (100 mg, 0.154 mmol), and NH_4OAc (700 mg, 9.09 mmol) were refluxed in methanol (6 mL) for 24 h. **12** precipitated as an off-white solid. It was filtered off, washed with methanol, and dried under vacuum. Yield: 11.4% (10.8 mg, 0.0176 mmol). ^{1}H NMR (500 MHz, 100 °C, TCE-d_2): δ 9.51 (2H, s, $H^{3''}$ or $H^{5''}$), 9.50 (2H, s, $H^{3''}$ or $H^{5''}$), 8.76 (2H, d, ^3J = 4.5 Hz, $H^{6''''}$), 8.59 (2H, s, H^3 and H^5), 8.59–8.55 (2H, m, $H^{3''''}$), 8.15–8.06 (6H, m, $H^{2'}$, $H^{6'}$, $H^{3'''}$, and $H^{5'''}$), 8.06 (4H, s, $H^{2'''}$ and $H^{6'''}$), 7.79–7.77 (2H, m, $H^{5''''}$), 7.66–7.63 (1H, m, $H^{4'}$), 7.62–7.57 (4H, m, $H^{3'}$, $H^{5'}$ and $H^{4''''}$); MALDI-TOF: calculated *m*/*z* for $C_{41}H_{27}N_7$: 619.66 $[M + 2H]^+$, found: 619.57; elemental analysis: calculated: %C 79.72, %H 4.41, %N 15.87; found: %C 80.14, %H 4.34, %N 15.81.

3,3’-PhSpPy (15).

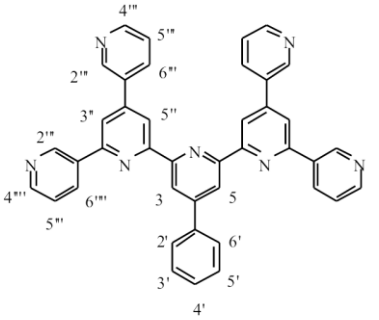

3,3'-Diazachalcone **11** (65.2 mg, 0.310 mmol), phenylpyridine bis(pyridinium iodine) salt **19** (100 mg, 0.154 mmol), and NH_4OAc (700 mg, 9.09 mmol) were refluxed in methanol (6 mL) for 24 h. **15** precipitated as an off-white solid. It was filtered off, washed with methanol, and dried under vacuum. Yield: 17.3% (16.4 mg, 2.66 mmol). ^{1}H NMR (500 MHz, 100 °C, TCE-d_2): δ 9.55 (2H, dd, ^5J = 0.5 Hz, ^4J = 2.0 Hz, $H^{2''''}$), 9.15 (2H, dd, ^5J = 0.5 Hz, ^5J = 2.5 Hz, $H^{2'''}$), 9.03 (2H, s, H^3 and H^5), 8.95 (2H, d, ^4J = 1.5 Hz, $H^{5''}$), 8.81 (2H, dd, ^4J = 1.5 Hz, ^3J = 5.0 Hz, $H^{4'''}$ or $H^{4''''}$), 8.78 (2H, dd, ^4J = 1.5 Hz, ^3J = 5.0 Hz, $H^{4'''}$ or $H^{4''''}$), 8.57 (2H, ddd, ^4J = 1.5 Hz, ^4J = 2.5 Hz, ^3J = 8.0 Hz, $H^{6''''}$), 8.17 (2H, ddd, ^4J = 1.5 Hz, ^4J = 2.5 Hz, ^3J = 8.0 Hz, $H^{6'''}$), 8.06 (2H, d, ^4J = 1.5 Hz, $H^{3''}$), 8.00–7.98 (2H, m, ($H^{2'}$ and $H^{6'}$) or ($H^{3'}$ and

$H^{5'}$)), 7.67–7.64 (2H, m, ($H^{2'}$ and $H^{6'}$) or ($H^{3'}$ and $H^{5'}$)), 7.60–7.58 (1H, m, $H^{4'}$), 7.55 (2H, ddd, ^{5}J = 0.5 Hz, ^{3}J = 5.0 Hz, ^{3}J = 8.0 Hz, $H^{5'''}$ or $H^{5''''}$), 7.53 (2H, ddd, ^{5}J = 0.5 Hz, ^{3}J = 5.0 Hz, ^{3}J = 8.0 Hz, $H^{5'''}$ or $H^{5''''}$); MALDI-TOF: calculated *m/z* for $C_{41}H_{27}N_{7}$: 619.66 $[M + 2H]^{+}$, found: 619.68; elemental analysis: calculated: %C 79.72, %H 4.41, %N 15.87; found: %C 79.40, %H 4.62, %N 15.76.

4,3’-PhSpPy (16).

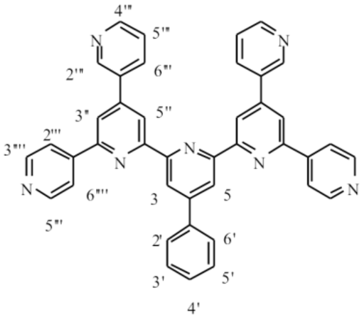

4,3'-Diazachalcone **8** (109 mg, 0.520 mmol), phenylpyridine bis(pyridinium iodine) salt **19** (158 mg, 0.243 mmol), and NH_4OAc (948 mg, 12.3 mmol) were refluxed in methanol (7 mL) for 24 h. **16** precipitated as an off-white solid. It was filtered off, washed with methanol, and dried under vacuum. Yield: 37.9% (57.0 mg, 0.0923 mmol). ^{1}H NMR (500 MHz, 100 °C, TCE -d_2): δ 9.15 (2H, d, ^{4}J = 2.0 Hz, $H^{5''}$ or $H^{2'''}$), 9.02 (2H, s, H^{3} and H^{5}), 8.99 (2H, d, ^{4}J = 1.5 Hz, $H^{5''}$ or $H^{2'''}$), 8.86 (4H, dd, ^{4}J = 1.5 Hz, ^{3}J = 4.5 Hz, $H^{3''''}$ and $H^{5''''}$), 8.82 (2H, dd, ^{4}J = 1.5 Hz, ^{3}J = 5.0 Hz, $H^{4'''}$), 8.19–8.16 (6H, m, $H^{6'''}$, $H^{3''''}$, and $H^{5''''}$), 8.11 (2H, d, ^{4}J = 1.5 Hz, $H^{3''}$), 8.00–7.98 (2H, m, ($H^{2'}$ and $H^{6'}$) or ($H^{3'}$ and $H^{5'}$)), 7.69–7.66 (2H, m, ($H^{2'}$ and $H^{6'}$) or ($H^{3'}$ and $H^{5'}$)), 7.62–7.58 (1H, m, $H^{4'}$), 7.57–7.54 (2H, m, $H^{5'''}$); MALDI-TOF: calculated *m/z* for $C_{41}H_{27}N_{7}$: 619.66 $[M + 2H]^{+}$, found: 619.52; elemental analysis: calculated: %C 79.72, %H 4.41, %N 15.87; found: %C 79.70, %H 4.75, %N 15.85.

STM

Zoomed STM images of 2,3’-PhSpPy (**13**) and 3,3’-PhSpPy (**15**):

a) b)

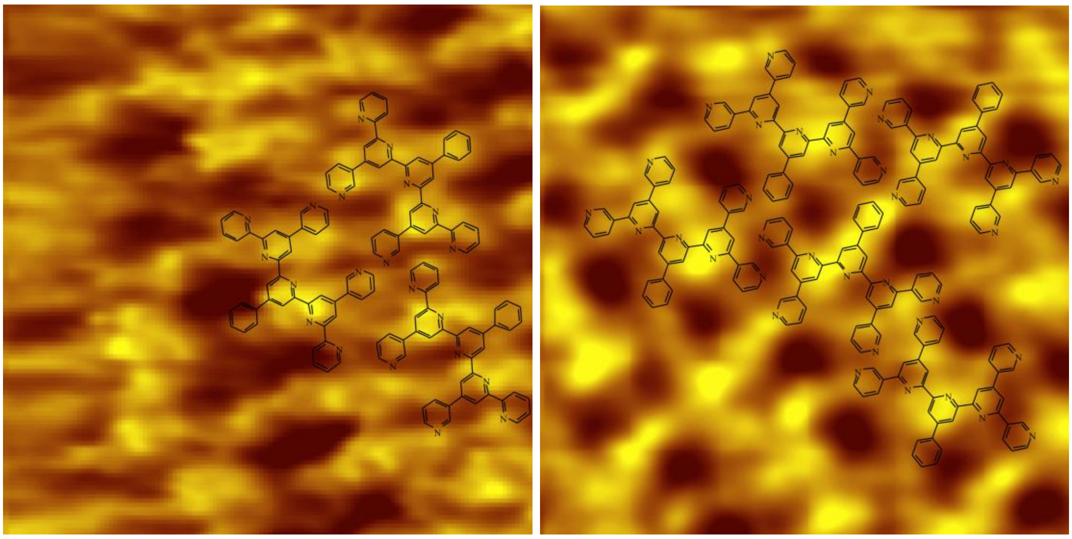

Figure S1: a) 5 × 5 nm^2 STM image (I_{set} = 2.10 nA, V_{set} = −600 mV) of 2,3'-PhSpPy (**13**) and b) 5 × 5 nm^2 STM image (I_{set} = 15.3 pA, V_{set} = −610 mV) of 3,3'-PhSpPy (**15**) at the HOPG/TCB interface with overlaid molecules.

References

1. Ziener, U.; Lehn, J.-M.; Mourran, A.; Möller, M. *Chem.–Eur. J.* **2002,** *8,* 951–957. doi:10.1002/1521-3765(20020215)8:4<951::AID-CHEM951>3.0.CO;2-9
2. Zhang, Z.; Dong, Y.-W.; Wang, G.-W. *Chem. Lett.* **2003,** *32,* 966–967. doi:10.1246/cl.2003.966
3. Durinda, J.; Szücs, L.; Krasnec, L.; Heger, J.; Springer, V.; Kolena, J.; Keleti, J. *Acta Fac. Pharm. Bohem.* **1966,** *12,* 89–129.
4. Soderquist, J. A.; Hsu, G. J.-H. *Organometallics* **1982**, *1*, 830–833. doi:10.1021/om00066a012
5. Perrin, D. D.; Armarego, W. L. F. *Purification of Laboratory Chemicals;* Pergamon: Oxford, 1998.
6. Meier, C.; Ziener, U.; Landfester, K.; Weihrich, P. *J. Phys. Chem. B* **2005**, *109*, 21015–21027. doi:10.1021/jp054271d
